# Radiation-induced melting in coherent X-ray diffractive imaging at the nanoscale

**DOI:** 10.1107/S0909049511016335

**Published:** 2011-05-26

**Authors:** O. Ponomarenko, A. Y. Nikulin, H. O. Moser, P. Yang, O. Sakata

**Affiliations:** a School of Physics, Centre of Excellence for Coherent X-ray Science, Monash University, Wellington Road, Victoria 3800, Australia; b School of Physics, Centre of Excellence for Coherent X-ray Science, University of Melbourne, Melbourne, Victoria 3010, Australia; c Department of Geological Sciences, The University of Saskatchewan, 114 Science Place, Saskatoon, Saskatchewan, Canada S7N 5E2; d Singapore Synchrotron Light Source, National University of Singapore, 5 Research Link, Singapore 117603; e Department of Physics, National University of Singapore, 2 Science Drive 3, Singapore 117542; f Institute of Microstructure Technology, Karlsruhe Institute of Technology, Postfach 3640, D-76021 Karlsruhe, Germany; g JASRI/SPring-8, Kouto 1-1-1, Mikazuki-cho, Sayo-gun, Hyogo 679-5148, Japan

**Keywords:** coherent X-ray diffraction imaging, high-resolution synchrotron radiation, heat load, nanosize effects

## Abstract

Coherent X-ray diffraction techniques play an increasingly significant role in the imaging of nanoscale structures, ranging from metallic and semiconductor to biological objects. In material science, X-rays are usually considered to be of a low-destructive nature, but under certain conditions they can cause significant radiation damage and heat loading on the samples. The qualitative literature data concerning the tolerance of nanostructured samples to synchrotron radiation in coherent diffraction imaging experiments are scarce. In this work the experimental evidence of a complete destruction of polymer and gold nanosamples by the synchrotron beam is reported in the case of imaging at 1–10 nm spatial resolution. Numerical simulations based on a heat-transfer model demonstrate the high sensitivity of temperature distribution in samples to macroscopic experimental parameters such as the conduction properties of materials, radiation heat transfer and convection. However, for realistic experimental conditions the calculated rates of temperature rise alone cannot explain the melting transitions observed in the nanosamples. Comparison of these results with the literature data allows a specific scenario of the sample destruction in each particular case to be presented, and a strategy for damage reduction to be proposed.

## Abstract 2

Coherent X-ray diffraction techniques play an increasingly significant role in imaging nanoscale structures which range from metallic and semiconductor samples to biological objects. The conventional knowledge about radiation damage effects caused by ever higher brilliance X-ray sources has to be critically revised while studying nanostructured materials.

## 1. Introduction

X-ray microscopy and coherent diffractive imaging (CDI; Miao *et al.*, 1999 ▶; Nugent *et al.*, 2003 ▶; Eisebitt *et al.*, 2004 ▶; Pfeifer *et al.*, 2006 ▶; Quiney *et al.*, 2006 ▶; Chapman* et al.*, 2006 ▶; Chapman & Nugent, 2010 ▶) and their modifications are rapidly developing as ultra-high spatial-resolution imaging techniques that exploit coherent, ultra-bright X-ray sources. To visualize an object at a nanoscale resolution, a significant amount of X-ray photons must be delivered to a very small volume. A modern synchrotron (*i.e.* 6–8 GeV third-generation machine) typically delivers approximately 10^10^–10^12^ photons  s^−1^ mm^−2^ at 8–20 keV within the coherence volume (Nikulin *et al.*, 2008 ▶). A 100 nm^3^ cube within the sample will scatter 10^2^–10^4^ photons per second at best. Since the diffracted intensity contrast is proportional to the product of the feature’s thickness and the refractive index difference at the boundary between sample and its environment, soft X-rays are much better suited to image materials with low electron densities (Sayre & Chapman, 1995 ▶; Chapman* et al.*, 2006 ▶). However, their use is limited owing to high vacuum requirements, so hard X-rays are preferred (Chapman *et al.*, 2006 ▶). The real part of the refractive index at ≥10 keV photon energy ranges between 10^−5^ and 10^−8^ in heavy metals and light elements, respectively, so that a much brighter source is required to visualize low-atomic-number samples at the true nanoscale, *e.g.* polymers or biological membranes with a spatial resolution of <10 nm (Chapman* et al.*, 2006 ▶; Nikulin *et al.*, 2008 ▶).

However, when the required density of photons increases as we approach a true nanoscale imaging, so does the radiation damage to the specimen (Sayre & Chapman, 1995 ▶). The ionizing nature of X-rays results in various damaging consequences to samples, which are serious limiting factors in macromolecular crystallography. Systematic studies on the dose dependence of specific types of radiation damage to certain classes of crystalline samples have been conducted. A so-called ‘Henderson limit’, *H* = 2 × 10^7^ Gy, introduced in macromolecular crystallography (Henderson, 1990 ▶), defines the dose at which the intensity of the diffraction pattern of a typical macromolecular crystalline sample is predicted to be halved. The macromolecular crystallography data consist of initially very strong peaks, which are Bragg reflections from a crystal lattice. The deterioration of the Bragg diffraction contrast is a result of many complex processes which happen within the macromolecular crystal during its X-ray exposure (Weik *et al.*, 2000 ▶). The primary effect of X-rays is the photoionization of preferentially core levels, followed by secondary processes like the emission of Auger electrons leading finally to conformational modifications of active centres, cleavage and re-arrangement of bonds (Weik *et al.*, 2000 ▶; Murray *et al.*, 2004 ▶; in ‘polymer language’ for PMMA, for instance, cleavage and re-arrangement correspond to main chain scission and cross linking) and heat.

In absorption-, transmission- and CDI-based X-ray microscopy of organic samples, radiation damage is widely acknowledged as a major problem and subjected to rigorous studies (Howells, Hitchcock & Jacobsen, 2009 ▶; Howells, Beetz *et al.*, 2009 ▶; Schafer *et al.*, 2009 ▶). The CDI schemes present an opportunity for the diffraction-limited three-dimensional structure determination of non-periodic objects, such as biological cells and nanocrystals. In practice, the resolution attained in CDI arises from a fine balance between fluence (the total number of photons per unit area) and dose (absorbed energy per unit mass; Howells, Beetz *et al.*, 2009 ▶; Marchesini *et al.*, 2003 ▶). In contrast to crystallographic diffraction, in the case of coherent diffractive imaging (Sayre & Chapman, 1995 ▶; Jacobsen & Kirz, 1998 ▶; Larson *et al.*, 2002 ▶; Chao *et al.*, 2005 ▶; Miao *et al.*, 1999 ▶; Nugent *et al.*, 2003 ▶; Eisebitt *et al.*, 2004 ▶; Pfeifer *et al.*, 2006 ▶; Quiney *et al.*, 2006 ▶; Chapman *et al.*, 2006 ▶), the data essentially represent a weak Fraunhofer diffraction pattern. For a given resolution, the non-periodic character of samples in CDI imposes more stringent conditions on coherence properties of the source and dose–fluence penalty relations (Howells, Beetz *et al.*, 2009 ▶; Marchesini *et al.*, 2003 ▶) than in conventional crystallographic schemes. However, from an analysis of maximum tolerable doses in both the CDI-based X-ray microscopy and macromolecular crystallography, Howells, Beetz *et al.* (2009 ▶) predicted that a particular feature of biological protein can be imaged with 10 nm resolution at a dose ∼10^9^ Gy. Based on the assumption that the material science samples have higher radiation tolerance, the authors (Howells, Beetz *et al.*, 2009 ▶) also predicted the possibility of coherent diffraction imaging of such samples with 1 nm resolution.

However, the assumption of higher tolerance to radiation damage of inorganic samples has to be tested for nano­structured materials. The physical properties of nanoscale materials differ from those in bulk owing to a larger surface/volume ratio and lower atomic coordination (Marks, 1994 ▶; Huang *et al.*, 2008 ▶). Noticeable effects of collective excitations (electronic confinement) also play an important role in the responses of nanostructured materials to external perturbations. These effects often result in the lower thermodynamic stability of nanomaterials in comparison with the bulk, and a spontaneous change of phase (*e.g.* quasimelting) has been observed even at low temperatures (Ajayan & Marks, 1988 ▶). For example, the quasimelting state of very small gold (∼1 nm) nanoclusters has been observed directly under an electron microscope (Marks, 1994 ▶). In CDXI experiments, even for larger nanostructures, the lowered stability could place serious limits on resolution owing to lowering the dose thresholds (Robinson, 2008 ▶; Marchesini *et al.*, 2003 ▶). However, there are almost no publications with quantitative data addressing the stability of material science nanosamples exposed to intense synchrotron radiation. Whether the Henderson limit is applicable for inorganic structures which do not contain carboxyl groups or sulfur bridges is an open question (Favre-Nicolin *et al.*, 2009 ▶). An important problem in X-ray diffraction studies is the temperature effect on the radiation dose tolerance. In biomolecular crystallography, cryocooling down to liquid-helium temperatures can prove to be advantageous against secondary radiation damage effects.

However, for electron tomography imaging of single frozen-hydrated biological objects such as large protein–membrane complexes, organelles and small cells with lower than atomic resolution (4–20 Å), a liquid-helium environment at 4–12 K did not provide any improvement in comparison with that of liquid nitrogen at ∼100 K (Iancu *et al.*, 2006 ▶; Bammes *et al.*, 2009 ▶). Systematic studies have shown that dose/damage relationships caused by either soft X-rays or electron beams in the polyethylene derivative samples are comparable (Wang *et al.*, 2009 ▶). Nevertheless, in X-ray imaging experiments the optimal experimental environment (*e.g.* high vacuum or a particular gas/liquid atmosphere, forced or natural convection) must be individually attuned with respect to the experimental method, sample material and target resolution.

In this paper we present experimental evidence for the destructive influence of synchrotron X-rays on nanoscale samples of both organic and metallic nature, show the role of heat loading in each case, and propose a tentative scenario to explain the observations.

## 2. Experiment

The experiments were performed at the BL13XU beamline at SPring-8, Japan. Synchrotron radiation energy of 12.4 keV was selected using a primary, tunable, double-crystal Si(111) beamline monochromator. Further angular collimation was performed using a double-crystal channel-cut Si(400) monochromator placed in non-dispersive mode. The beam was then spatially collimated by two pairs of slits defining a 0.3 mm (height) × 0.2 mm (width) beam incident on the sample. Samples were placed on a linear motion stage downstream immediately beyond the slits in such a way that the X-ray diffraction from it occurred in the vertical plane coinciding with the diffraction plane of the X-ray optics. A Si(400) crystal analyzer and a scintillation detector were placed downstream from the sample to collect the diffracted intensity from the sample as a function of the angular position of the analyzer. The sample was then scanned across the collimating slits to expose different nanostructures to the incident beam. The experimental chamber was kept under ambient conditions, *e.g.* the sample was cooled by natural convection of air under normal pressure and room temperature. Samples of known geometry composed of 200 nm-thick PMMA resist were deposited on ∼5 mm × 5 mm-wide 1 µm-thick Si_3_N_4_ membranes held by a thicker silicon window-frame and consisted of 3 × 3 fields of 500 µm × 500 µm areas, which were filled with various patterns including holes, posts and lines and spaces. The characteristic pattern sizes were 100, 200 and 500 nm. We also examined a sample which included 50 nm-diameter gold nanoparticles which were dispersed densely, but not uniformly, in a 1 µm gap between two 50 µm-thick kapton sheets. The estimated volume fraction filled by gold nanoparticles was ∼45–50%.

## 3. Results

### 3.1. Damage state of nanosamples

**Figure 1:**
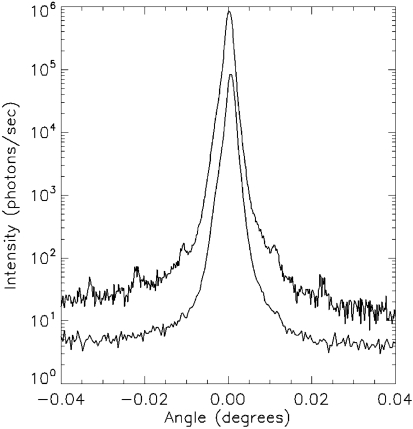
Fraunhofer diffraction profiles recorded from a test nanostructure in PMMA resist at the BL13XU beamline, SPring-8, Japan: the upper and lower curves were recorded under the same experimental conditions with an interval of approximately 20 min. The curves are shifted by an order of magnitude for better visibility.

**Figure 2:**
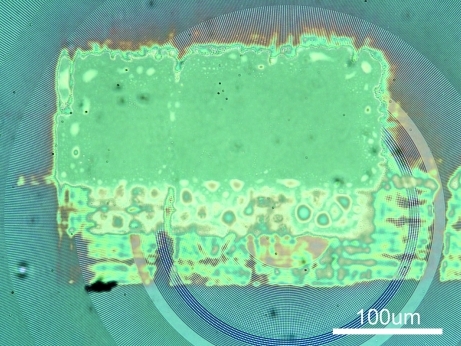
Photograph of the sample with test nanostructures produced in PMMA resist after the experiment.

**Figure 3:**
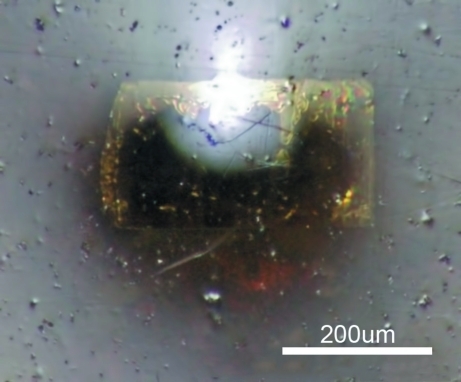
Photograph of the sample with gold 50 nm-diameter nanoparticles dispersed between kapton sheets after the experiment. The ‘solid’ gold area corresponds to the size of the incident beam.

Whilst searching for the best position to record experimental data suitable for phase-retrieval reconstruction of the kapton–gold sample, we briefly observed a few diffraction patterns with satellite peaks positioned on both sides of the central reflection from the crystal analyzer (Fig. 1 ▶). However, no repeated scan on the same spot within the sample showed the presence of those diffraction patterns any longer. Further examination of the sample showed that the areas exposed to the X-ray beam had the gold nanostructures destroyed completely or almost completely (Fig. 2 ▶). An attempt at image-dispersed gold nanoparticles resulted in a totally unexpected diffraction pattern that could not be explained using *a priori* knowledge of the sample. Further examination of the sample showed that the area exposed to the X-ray beam had transformed into a solid gold film (Fig. 3 ▶), so the samples lost their structural integrity while being irradiated by X-rays. While we can assume broken bonds or chain scission as a result of radiation damage in the case of PMMA samples, the gold nanoparticles have undergone a severe melting transition.

## 4. Heat load and damage mechanisms

### 4.1. Classical heat-load model

**Figure 4:**
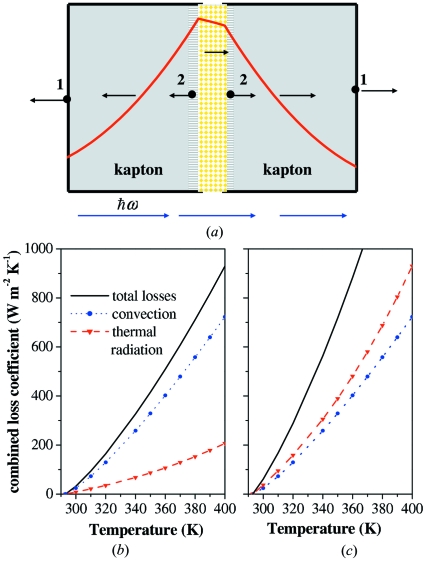
(*a*) Schematics of heat-flow directions in the model of a sample with gold nanospheres dispersed with a 1 µm gap between 5 µm-thick kapton sheets. The blue arrows denote the direction of the X-ray beam. The red line denotes the temperature distribution in the sample. The outer boundary points are labeled with 1 and the points at the interfaces of the material are labeled with 2. (*b*) Temperature dynamics of the thermal loss coefficient for the surface with emissivity ∊ ≃ 0.2 marked by triangle symbols, the convection coefficient [equation (5) in the text] marked by circles, and the combined (convection + thermal radiation loss) coefficient marked by a solid line. (*c*) Same as (*b*), with ∊ ≃ 0.9.

In this series of experiments the detailed temperature measurements of the sample and environment were not conducted. We employed numerical simulations to estimate the heat-loading regimes in the composite samples. Photoelectric absorption plays the most significant role compared with Rayleigh and Compton scattering for light and moderately heavy *Z* elements interacting with photon energies well below 100 keV. The heating results from the X-ray-induced excitation of electrons in the material and a subsequent transformation of their energy into lattice phonons. The photoelectron emission is accompanied by radiative or Auger relaxation, which is followed by cascades of secondary (δ) electrons. Photo-, Auger- and secondary electrons are thermalized through multiple collisions (Beloshitsky *et al.*, 1993 ▶; Holmes-Siedle & Adams, 1994 ▶; Attix, 2004 ▶; Ocola & Cerrina, 1993 ▶). The photon energy deposited in the material can be found using the photon mass-energy absorption coefficient (Hubbell, 1982 ▶; Henke *et al.*, 1993 ▶). We adopted the ‘heat balance model’, which was used previously in simulations of mask deformations induced by radiation heating in X-ray lithography (Chiba, 1992 ▶). Inside the material (sample or support) the heat is generated due to photon absorption and transferred through the material *via* conduction, and the outer surfaces lose energy due to convection and radiative transfer mechanisms; see Fig. 4 ▶(*a*) for the schematics of heat flows. A one-dimensional finite difference scheme (Holman, 2002 ▶) was employed to approximate the general heat balance equation
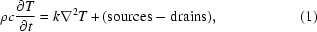
with boundary conditions
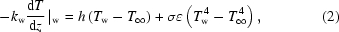
where subscript w represents ‘wall’, σ is the Stephan–Boltzmann constant, ∊ is the surface emissivity, and *T*_∞_ is the temperature of the environment. Inside the material the governing heat-balance equation (1) is
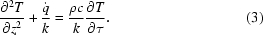
The heat source 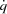 due to incident photon energy at the inner node *m* for a layer of a homogeneous material can be calculated as
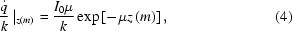
where μ is the material-dependent attenuation coefficient (Hubbell, 1982 ▶; Henke *et al.*, 1993 ▶) evaluated for the experimental value of a photon energy for 0.1 nm wavelength X-rays, *E* = 2 × 10^−15^ J (∼12.4 keV). The X-ray beam energy-density rate, *I*_0_ = *FE*, was calculated for the flux *F* of 6.3 × 10^13^ photons s^−1^ cm^−2^, which was measured using a PIN diode detector. The material-dependent parameters used in the simulations are listed in Table 1 ▶.

For each sample the height and width (along *x* and *y* directions, respectively) were much larger than the thickness of the sample. The propagation of heat was considered only along the beam (*z* direction). This simplified model was aimed at assessing the overall heating dynamics of the samples for the extreme case, *i.e.* for continuous exposure without conduction losses in the lateral dimensions. The calculated temperature distributions in the samples appeared to be very sensitive to the simulation parameters, in particular to the coefficient of free convection *h* and the radiation heat-transfer emissivity ∊ at the surfaces contacting with the ambient air, see equation (2). The coefficient of free convection was calculated using approximations for a vertical plane of a varied height of the external surface of the sample (Holman, 2002 ▶), and a simplified expression for free air convection in ambient conditions,
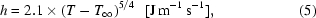
taken from Carslaw & Jaeger (1947 ▶). The value of the contact resistance (Holman, 2002 ▶) at the boundary between different materials was also an important parameter influencing the heat-transfer dynamics, particularly for the sample consisting of gold nanospheres placed between two vertical kapton sheets of 50 µm thickness. At the first stage of the simulations, the gold sample was modeled as a 1 µm film of gold. The important effects of nanoporosity on the heat transport properties and the way of embedding them in the heat transport model are discussed in §4.3.

For the experimental parameters of the beam we also simulated the heat loading in a two-layered structure consisting of a 200 nm layer of PMMA placed on top of a silicon nitride support with varying thickness of 1–50 µm.

### 4.2. Modeling of heat load in PMMA nanosamples

The major heat losses in the PMMA–Si_3_N_4_ system, modeled as a free-standing double layer, are:

(i) Heat losses owing to heat radiation transfer,
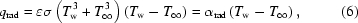
where 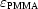 ≃ 0.9 and 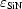 ≃ 0.2 (Table 1 ▶).

(ii) Free-convection losses, where *h* is given by equation (5),[Disp-formula fd5]

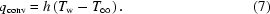
The heat losses increase nonlinearly with temperature, as shown in Figs. 4 ▶(*b*) and 4(*c*). At the temperatures close to the ambient conditions, the largest losses occur due to the convection cooling at the surface of PMMA, and, to a lesser extent, at the outer surface of Si_3_N_4_. The role of thermal radiation losses increases as *T* ^4^ and dominates at elevated temperatures, particularly for surfaces with higher emissivity, see Fig. 4 ▶(*c*).

The difference between thermal diffusivity values at the polymer–support interface, α_PMMA_ ≃ 2 × 10^−7^ m^2^ s^−1^ and α_Si3N4_ ≃ 10^−5^ m^2^ s^−1^ (where α = *k*/*c*ρ), may result in a noticeable thermal contact resistance on the boundary between these materials. To estimate the interface heat conduction for various values of surface roughness, we utilized simplified expressions for heat contact resistance (Holman, 2002 ▶),
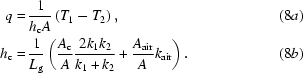
Here *k*_*i*_ (*i *= 1, 2, air) are coefficients of conduction for silicon nitride, PMMA and air, respectively, *h*_c_ is the contact resistance, *A* is the total area at the interface, *A*_c_ is the contact area, *A*_air_ is the area of ‘void’, and *L*_g_ is the width of a ‘gap’ between the two materials. Within the framework of the heat-transfer model, even for a small contact area at the interface, the simulated temperature difference between the two materials stabilizes within a few time steps, *i.e.* several picoseconds. However, the thermal resistance models which are based on macroscopic parameters are not reliable at the nanoscale. Also, to account for the sub-nanometer feature size at the interface of materials, a much shorter time step size of the order of a femtosecond must be chosen. However, Fourier’s law is inapplicable even at length scales larger than the phonon mean free path in nanostructures (tens of nanometers; Hopkins *et al.*, 2011 ▶). At these time and spatial scales, ultrafast spectroscopy, molecular dynamics and statistical mechanics models (phonon mismatch models) have to be used to account for the thermal boundary conductivity.

In the PMMA–Si_3_N_4_ system the X-ray-induced temperature rise occurs mainly due to the silicon nitride support. The heat generation in a thinner layer of PMMA can be neglected due to the very low X-ray photon absorption of PMMA (Table 1 ▶) and much lower (two orders of magnitude) thermal diffusivity coefficient α = *k*/*c*ρ compared with silicon nitride. Therefore, for our purposes we can approximate this system by a single layer of Si_3_N_4_. An analytical steady-state solution of equation (1) with the boundary conditions given in equation (2) for a single Si_3_N_4_ layer was propagated iteratively in time. If the time steps are sufficiently small, the iterative solution accurately reproduces the results of the direct transient finite-difference scheme, providing a much more efficient computational alternative to the latter.

**Figure 5:**
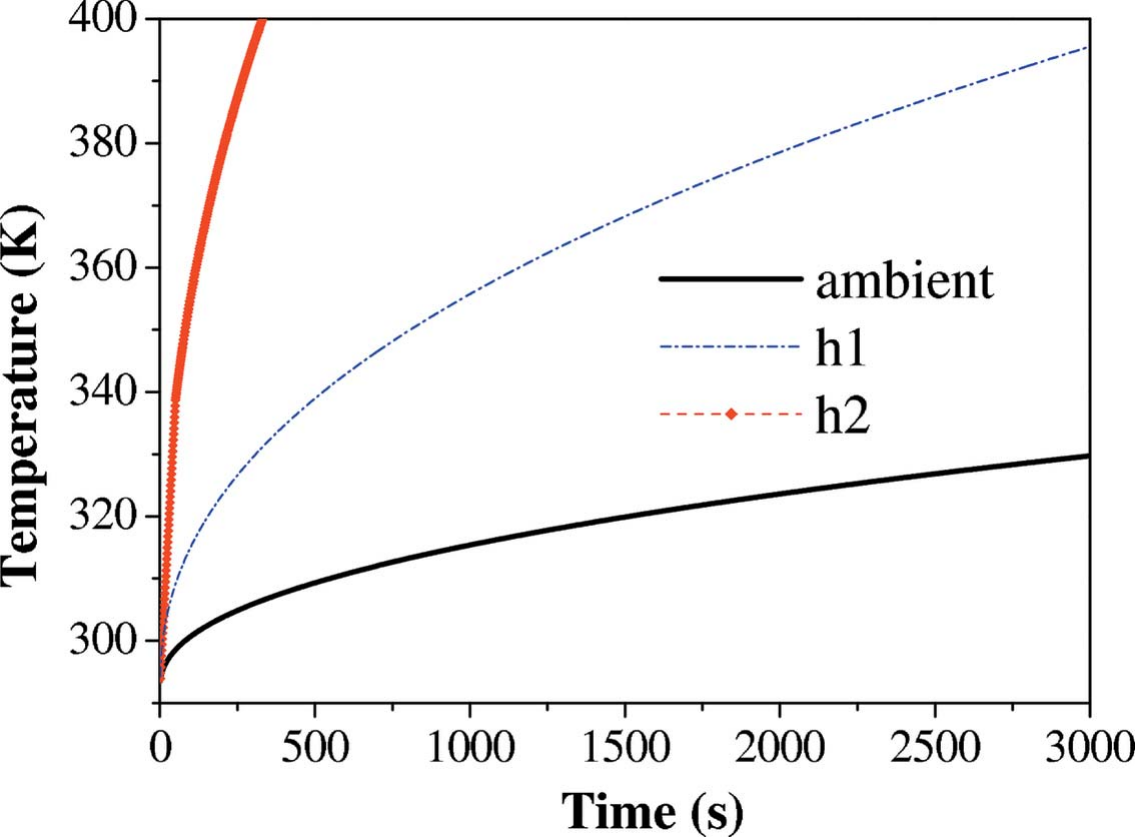
Prolonged simulation of the maximal temperature growth for the 1 µm-thick slab of Si_3_N_4_ support for different parameters of heat convection and thermal radiation emissivity ∊. The solid line denotes the ambient conditions with surface emissivity coefficients, ∊_SiN_ = 0.2, ∊_PMMA_ = 0.9, the dot–dashed line denotes conditions with emissivity ∊_SiN_ = 0.02, ∊_PMMA_ = 0.09 and heat conductivity *h*1 = 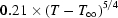
 J m^−2^ s^−1^; the dashed line with diamond marks denotes conditions with heat conductivity *h*2 = 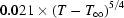
 J m^−2^ s^−1^ and ∊ = 0.002.

Our experimental results showed that a nanostructure made of PMMA resist can completely lose its structural integrity within 10–20 min. The melting temperature for PMMA ranges between 343 and 438 K depending on the molecular mass (Ute *et al.*, 1995 ▶) and previous radiation damage (El-Kholi *et al.*, 2000 ▶). We simulated different regimes of heat-loss processes such as thermal radiation to the environment and convection cooling, described by emissivity, ∊, and the coefficient of heat convection, *h*, respectively, for a 200 nm-thick nanolayer of PMMA on a 1 µm-thick silicon nitride support. For the almost suppressed convection [*h*2 = 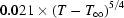
 J m^−2^ s^−1^] and negligible emissivity of the outer and back sample surfaces (∊_PMMA_ = ∊_SiN_ = 0.002), the simulated temperature of the sample, 393 K, was attained after ∼4.7 min of exposure. Increased emissivity values (∊_SiN_ = 0.02, ∊_PMMA_ = 0.09) and a higher convection rate [*h*1 = 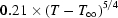
 J m^−2^ s^−1^] slowed down temperature growth, so the rise from 293 to 393 K happened after 47.3 min of exposure. However, for realistic parameters of heat-loss parameters [∊_SiN_ = 0.2, ∊_PMMA_ = 0.9 and ambient convection described by equation (5)] the simulated final temperature for this sample is much lower than the melting threshold for PMMA resist. Our calculations predicted that, after 1 h of continuous exposure of the sample to X-ray radiation under ambient conditions, its temperature will grow only moderately, by ∼30° (Fig. 5 ▶). Also, to estimate the contribution of the silicon window frame to the heat load of the sample, we conducted simulations for a 1 µm silicon layer. For the realistic heat conduction and standard thermo-physical parameters of silicon (Table 1 ▶), the simulated temperature of the layer did not increase by more than 40–45°. Since the beam spot size in the experimental set-up (0.2 mm × 0.3 mm) was smaller than the size of the membrane covered by the polymer (0.5 mm × 0.5 mm), the additional heat load on the sample due to the X-ray exposure of the silicon frame should not be significant.

These simulations provided ‘the worst-case scenario’, when the sample is continuously exposed to X-rays. However, the real experiment was conducted in a scanning mode by pulsed synchrotron radiation, which usually results in a lower temperature rise (Heinrich *et al.*, 1983 ▶). Therefore, to explain the experimentally observed degree of damage inflicted on the PMMA samples by synchrotron radiation, we have to look at the deeper mechanisms underlying interactions of organic polymers with X-rays.

### 4.3. Heat-load and heat-loss mechanisms in gold nanoparticles

In our heat-transfer simulations we modeled a layer of 50 nm gold nanoparticles dispersed in a 1 µm gap between kapton sheets as a 1 µm layer of solid gold. In doing so we neglected the radiation transfer losses between gold nanospheres, which could potentially contribute to a higher temperature rise of the nanosample. However, since the gold nanoparticles were assumed to be closely packed inside the gap, the heat-conduction transfer processes within the gold sample would dominate under ambient temperatures. Since the X-ray attenuation coefficient for kapton is very small (Table 1 ▶), the largest part of the absorbed photon energy is deposited in the gold film and transferred *via* heat conduction through the kapton layers, and then lost to the environment *via* free convection and heat radiation. Under ambient conditions the convection and radiation losses are described by equations (6) and (7) which take into account the emissivity of kapton (∊ ≃ 0.9). The contact resistance at the interface between the gold layer and kapton sheets for varied contact areas was calculated using equations (8*a*) and (8*b*). However, in this model the effect of contact resistivity on the rate of temperature rise was far less important than the heat convection and radiation losses.

For this sample we have tested a range of convection and radiation transfer parameters and were able to identify different regimes of the heat-transfer dynamics.

**Figure 6:**
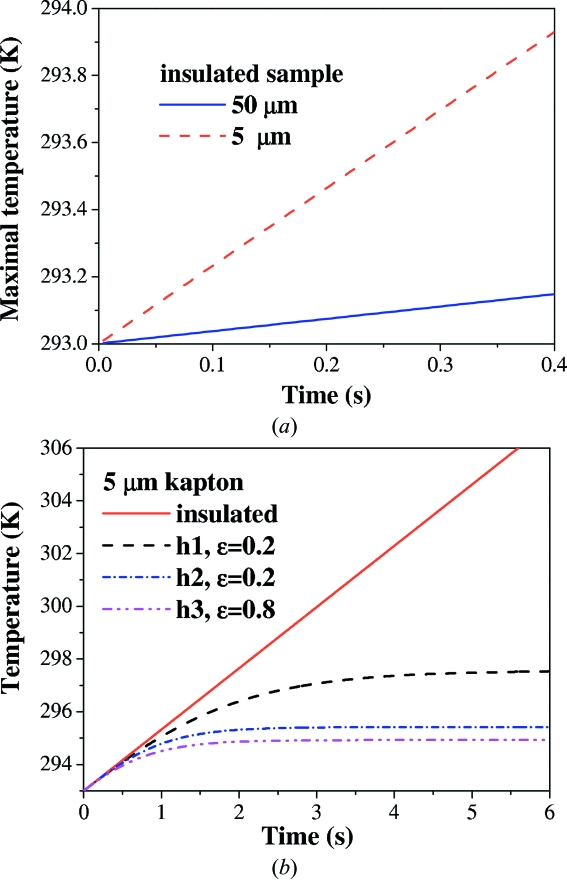
Results of numerical simulations of 50 nm gold nanospheres dispersed in 1 µm gaps between thick kapton sheets. (*a*) Maximal temperature growth rate for insulated samples with different thickness of kapton sheets. The dashed line denotes results for 5 µm kapton sheets, the solid line for 50 µm-thick kapton sheets. (*b*) Maximal temperature growth in the sample with 5 µm kapton sheets for different parameters of heat conduction and emissivity ∊. The solid line denotes the insulated sample, the dashed line denotes conditions with emissivity ∊ = 0.2 and heat conductivity *h*1 = 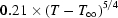
 J m^−2^ s^−1^; the dot–dashed line denotes conditions with heat conductivity *h*2 = 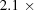 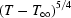
 J m^−2^ s^−1^ and ∊ = 0.2; the dot–dot–dashed line denotes heat conductivity *h*3 = *h*2 as above and emissivity ∊ = 0.8 (*i.e.* ambient conditions).

(i) The maximum temperature in the sample for the experimental beam parameters and the realistic (ambient) values of the free air convection and emissivity never increased *by more than a few degrees* above the initial ambient temperature and quickly reached saturation (see Fig. 6 ▶ for comparison of temperature rise in gold samples with different kapton thickness).

(ii) For higher values of convection (∼100 W m^−2^ K^−1^) and high surface emissivity (0.9–1) the temperature distribution in a sample attained a saturation regime after rising *for a fraction of degree* in a matter of tenths of a second.

(iii) For suppressed convection (*h* < 0.0001 W m^−2^ K^−1^) and suppressed surface emissivity, the melting temperature of bulk gold, *T* = 1336 K, in the kapton–gold–kapton sample was achieved after ∼40 min. However, in the extreme case of a fully insulated 1 µm film of gold (*i.e.* without kapton layers), the bulk melting temperature of gold was attained after slightly more than 6 s of exposure. [The melting temperature grows with size of nanoparticles, and for gold nanoparticles of >20 nm it quickly approaches bulk values, so the estimation using dependence from the textbook by Buffat & Borel (1976 ▶), gives a melting point of ∼1130 K for 50 nm-diameter gold nanospheres.]

The role of the thickness of the insulating material (kapton) has also been studied. We repeated simulations for a 1 µm film of gold placed between two thin (5 µm) kapton layers. The respective graphs of maximum temperature rise for different parameters of heat conduction and emissivity for this system are shown in Fig. 6 ▶(*b*). The effect of kapton thickness for suppressed convection and negligible emissivity is shown in Fig. 6 ▶(*b*). The temperature growth rate for a sample with 50 µm-thick kapton sheets is ∼0.2 K s^−1^; however, for a sample with 5 µm kapton layers it is much higher, ∼2.3 K s ^−1^. Even a moderately thick layer of a low-absorbing material like kapton decreases the heat growth rate considerably even for suppressed heat losses (Fig. 6 ▶ *a*). The thermo-insulated system with thin kapton layers attains the melting temperature of gold after 7.5 min of exposure (∼6 min to reach the reduced melting point of ∼1130 K). The largest part of the heat is generated by the photon energy absorption in the gold layer. However, the spatial temperature gradients within the sample are small due to the high conductivity of the boundary layer for the simple model of the surface contact resistance (Holman, 2002 ▶) between gold and kapton layers.

While we were able to tune up the numerical parameters in the classical heat-transfer model and to simulate the generation of the bulk melting temperatures in the kapton–gold sample within minutes of exposure to X-ray beam, these parameters (*i.e.* corresponding to suppressed free convection and low emissivity) do not reflect the typical experimental conditions. Careful monitoring of temperature distributions in the sample and its environment during experiments is necessary to improve our understanding of heat-loading processes. For our microscopically thin samples the heating rate is quite sensitive to convection, ‘black-body’ radiation and conduction losses. The temperature rise slows down noticeably when realistic heat sinks are introduced in the model. We have to look for additional explanations of the fast (within 10–20 min) melting transition of gold nanospheres observed in our experiments. One such effect, which can be easily included in macroscopic heat-transfer simulations, is the change of thermal conductivity in nanostructured materials.

Thermal conductivity in nanoporous materials can be reduced substantially owing to the electron-surface scattering and reduction of the electron mean free path. If the pores are treated as randomly sized spheres, the reduction in thermal conductivity of the porous film may be estimated by (Hopkins *et al.*, 2008 ▶)
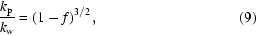
where *f* is the porosity (volume fraction of air in the material), *k*_w_ is the already reduced thermal conductivity of the solid dense non-homogeneous material (in our case it is the reduced thermal conductivity owing to scattering from the boundary of gold nanospheres), and *k*_p_ is the reduced thermal conductivity of the nanoporous Au. The reduction in electronic thermal conductivity associated with particle boundary scattering is given by (Hopkins *et al.*, 2008 ▶)
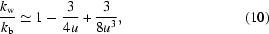
where *k*_w_ is the reduced thermal conductivity, *k*_b_ is the conductivity of the corresponding bulk material, and *u* is the ratio of the particle diameter, *d* = 50 nm, to the electron mean free path in the wire, λ ≃ 45 nm. Even for a high density of particles (porosity *f* = 0.1), the thermal conductivity *k*_p_ ≃ 66.9 W m^−1^ K^−1^ calculated from equations (9) and (10) is substantially lower than the ambient bulk conductivity of gold, *k*_b_ = 310 W m^−1^ K^−1^. However, porosity will decrease the effective mass attenuation coefficient owing to the lower density of a solid material,
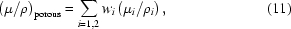
where *w*_*i*_ is the fraction by weight, ρ_*i*_ is the density and μ_*i*_ is the mass attenuation coefficient of the *i*th material constituent. The heat generated as a result of X-ray absorption in a porous material will therefore depend on a balance between the decreased absorption of photon energy and the higher heat accumulation rate owing to the reduced thermal conductivity. Since the mass-energy absorption coefficient and density are much higher in gold than in air, the porosity will lead to a slight decrease in the X-ray photon energy absorption, *e.g.* for porosity *f* = 0.1 the effective mass absorption coefficient will be reduced by only 10%, while reduction of thermal conductivity will amount to almost 80%. Similar calculations for our sample (50 nm gold nanoparticles dispersed between kapton sheets) characterized by *f* = 0.5 show that the decrease in thermal conductivity will be ∼96%, while the effective mass absorption will be reduced by less than 50%. As a result, the heat generated *via* X-ray photon absorption will be accumulated much more efficiently in the nanoporous gold sample than in a solid film of gold. This is just one example of the role played by nanoscale effects in the X-ray-induced heat loading. Here we neglect the ‘thermal bath’ effects generated by the thermal radiation transfer between the gold particles.

In the following section we briefly review features of the nanoscale thermal transport and its effects on X-ray radiation-induced damage in heterogeneous nanostructures.

## 5. Nanoscale effects

### 5.1. Applicability of classical heat-load model at nanoscale

In studies of radiation heating effects in nanolithography, it has been noted that macroscopic heat-transfer models are not accurate enough to predict temperature rises during exposure of micro- or nanosized objects to intense synchrotron radiation (Vladimirsky *et al.*, 1989 ▶). Indeed, the classical heat-transfer laws are based on the assumption of an instantaneous response of the system to changes in the supplied heat which is not valid at the nanoscale (Volz, 2007 ▶). Numerical simulations of heat conduction in complex nanomaterials require fine spatial grids and, correspondingly, very short time steps (∼10^−11^–10^−18^ s, depending on model parameters). Unfortunately, the dynamics of many atomistic processes, which are important on these time and spatial scales, is not captured by the classical heat-transfer models. The sizes of nano-objects are comparable with characteristic lengths of the heat generation and conduction processes, such as electron or phonon mean free paths. This in turn affects the temporal limits of applicability for classical thermal diffusion models. For example, for a typical value of the thermal diffusivity coefficient, α = *k*/*c*ρ ≃ 10^−6^ m^2^ s^−1^, an estimated time needed to attain a homogeneous temperature of a 1 nm-diameter particle heated at the surface is ∼1 ps, which is of the same order as the phonon relaxation time.

A number of fast coupled processes are important in the radiation-induced heating of nanostructures:

(i) Single-event ionization processes that happen on a timescale of attoseconds (Krausz & Ivanov, 2009 ▶);

(ii) Thermalization of electron distribution which is of the order of 1 fs to a few hundred femtoseconds (Allen, 1987 ▶).

(iii) Equilibration of energy transferred from the electron system to the phonon lattice (electron-phonon coupling) which happens on a timescale of a few picoseconds (Allen, 1987 ▶; Schafer *et al.*, 2002 ▶).

(iv) Phonon–phonon relaxation time, which is responsible for cooling due to heat transfer at the interfaces of materials, which is of the order of hundreds of picoseconds (Link & El-Sayed, 1999 ▶) and, in some cases, nanoseconds (Wu *et al.*, 2007 ▶; Highland *et al.*, 2007 ▶).

Analysis of the timescales involved in radiation-induced heating shows the bottlenecks of the heat-transfer processes. For example, for moderate energy-deposition rates in nano­structures, as in our case, inefficient cooling at the interfaces of materials (sample–air, sample–support) may prove detrimental. Underestimation of the contact resistance at the interface between materials may result in lower simulated temperature rises, compared with real nanomaterials. The presence of large interface areas in complex nanostructured materials changes not only heat conduction, but also thermodynamic stability and photon scattering compared with those properties in the bulk. These effects are discussed in the following section.

### 5.2. Nanosize effects and role of interfaces in X-ray-induced phase transitions

Nanosize effects may enhance radiation damage by changing energy deposition, heat conduction and phase stability properties of irradiated materials in multiple ways. For example,

(i) Decreased thermal heat conductivity in nanoporous materials, as discussed above;

(ii) Enhanced emission of photo- and secondary electrons in nanoparticles and plasmonic losses;

(iii) Changes in the thermodynamic stability of nano­structured materials.

The *energy deposition* pathways resulting from X-ray impact on heterogeneous nanomaterials are complex. The primary photoelectrons released in interactions with 0.1 nm X-ray radiation are very fast, with the initial velocity over 60 nm fs^−1^ (Ziaja *et al.*, 2005 ▶). Such electrons have enough energy to escape from one nanostructure and penetrate into another if the collection of nanoparticles is ‘closely packed’. The kinetic energy of a primary photoelectron is reflected in its range and in the radius of the energy-deposition region owing to secondary electron cascades (Ziaja *et al.*, 2005 ▶). The number of escaped electrons (photoelectron yield) is increased in nanoparticles (Lewinski *et al.*, 2009 ▶). Improvement of the detailed resolution in X-ray lithography (Han *et al.*, 2002 ▶), development of new detector materials for XFEL (X-ray free-electron laser) experiments (Gabrysch *et al.*, 2008 ▶) and medical applications, such as gold nanoparticle-aided X-ray radiation therapy (Jones *et al.*, 2010 ▶), have prompted detailed investigations of the spatio-temporal dynamics of secondary electron cascades (Gabrysch *et al.*, 2008 ▶); however, the low-energy electron cross-section data needed for atomistic Monte Carlo simulations still lack accuracy (Ziaja *et al.*, 2005 ▶). A large part of the photon energy deposited in materials *via* low-energy secondary electron cascades is attributed to plasmonic losses (Ritsko *et al.*, 1978 ▶; Dapor *et al.*, 2010 ▶; Han *et al.*, 2002 ▶). However, the role of plasmonic effects in the total heat loss is difficult to quantify for X-ray photoemission regimes.

The thermodynamic stability of nanostructured materials differs from that of the bulk (Kelly *et al.*, 2003 ▶; Marks, 1994 ▶; Allen *et al.*, 1986 ▶; Cahn, 1986 ▶; Buffat & Borel, 1976 ▶). Small free-standing metal nanoparticles exhibit a decrease in melting point which is inversely proportional to their size (Buffat & Borel, 1976 ▶; Allen *et al.*, 1986 ▶; Marks, 1994 ▶). Thermodynamic stability is influenced by the shape of the particles, but these effects may differ for free-standing and colloidal nanoparticles (Barnard *et al.*, 2005 ▶; Allen *et al.*, 1986 ▶; Link & El-Sayed, 2000 ▶). The mismatch in physical properties at the surface boundaries between different materials (*e.g.* metals and dielectrics) drives such effects as wetting (Lipowsky, 1990 ▶). Surface effects, including the effects of confinement, influence thermodynamic stability in a number of ways. Depending on the environment, nanoparticles may show decreased or increased melting temperature as well as various phase transition effects (Alba-Simionesco *et al.*, 2006 ▶). For example, nanoparticles embedded *via* annealing in a matrix of a host material with a higher melting point also show an increased melting temperature (Mei & Lu, 2007 ▶). However, embedding nanoparticles of low-melting-point materials in a confined space may prevent their crystallization (Kobayashi *et al.*, 2010 ▶). External perturbations, such as electron or ion irradiation or ball milling may lead to structural changes and alloying at low temperatures. Such dynamic changes of nanosized materials are described by a concept of ‘driven materials’ (Bellon & Averback, 2003 ▶). In response to external perturbations, the surfaces of nanoparticles are the first to show signs of state transitions, so-called ‘surface melting’ (Cahn, 1986 ▶; Peters *et al.*, 1997 ▶, 1998 ▶). This effect has been observed at surface temperatures much lower than the bulk melting temperature.

In addition, if a material undergoes chemical modifications caused by X-ray irradiation, as in organic polymers, it is the interfaces between ‘sample–support’ and ‘sample–atmosphere’ that accumulate the largest number of defects.

## 6. Radiation damage mechanisms in nanosamples

### 6.1. Damage scenario for PMMA

Photoionization and multiple excitations due to secondary electron collisions in irradiated PMMA result in bond breaking (chain scissions), accompanied by the generation of reactive, short-lived intermediate compounds, in particular, free radicals. Exposure to ambient oxygen and water increases the degree of chemical degradation in polymers due to oxidation and free-radical formation. Radiochemical reactions in PMMA following photoionization-induced scissions include a mixture of complex reactions, such as cross-linking, recombinations, disproportions, rearrangements, transfer reactions and out-gassing of CO, CO_2_, H_2_ and CH_4_ gases, all resulting in mass loss (Schmalz *et al.*, 1996 ▶; Coffey *et al.*, 2002 ▶). Prolonged exposure of PMMA to ionized radiation causes its decomposition into shorter chains (monomerization) and consecutive lowering of the melting temperature, so that the irradiated polymer may eventually liquefy (Holmes-Siedle & Adams, 1994 ▶). In industrial applications, PMMA is used below its glass transition temperature (*T*_g_) which lies between 373 and 398 K, depending on the composition (Schmalz *et al.*, 1996 ▶). Transition from glassy to viscous state involves excitations of vibration movements in the polymer backbone chain generated by input of a thermal or electromagnetic energy. The temperature of melting (*T*_m_) defined by the transition from crystalline to liquid state of PMMA is higher than its *T*_g_. Similar to the majority of polymers, the relation between *T*_m_ and *T*_g_ in PMMA is approximately linear; however, the exact ratio of *T*_m_/*T*_g_ depends on a number of factors (van Krevelen & te Nijenhuis, 2009 ▶). Both *T*_m_ and *T*_g_ can be changed by chemical modifications in the backbone of the polymer. In particular, a strong depression of both *T*_g_ and *T*_m_ is observed on decreasing the degree of polymerization (Ute *et al.*, 1995 ▶). The thermodynamic stability of PMMA nanostructures is strongly affected by the confinement and interface interactions (Keddie *et al.*, 1994 ▶; Forrest & Dalnoki-Veress, 2001 ▶; Moller *et al.*, 1998 ▶; Rittigstein & Torkelson, 2006 ▶). These observations are important for analyzing the phase transitions of PMMA under intense X-ray radiation.

Depending on the research field, in the literature a complicated terminology exists defining the degree of radiation impact on material. To avoid confusion, in this work a standard kerma dose definition expressed in Gray (Gy) units was used. By definition, the kerma (*K*) dose is a kinetic energy of radiation released in the material assuming that all the energy absorbed in the material is converted into the dose (Holmes-Siedle & Adams, 1994 ▶; Attix, 2004 ▶),
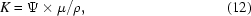
where Ψ is the radiation energy fluence and μ/ρ is the mass-transfer coefficient for the material (1 Gy equals 1 J kg^−1^, *i.e.* the amount of deposited energy in J kg^−1^ of sample material). In the literature there is a considerable discrepancy in the threshold dose values for melting and glass transition temperatures of irradiated PMMA (Silva *et al.*, 2010 ▶; Coffey *et al.*, 2002 ▶; Schwahn & Gesell, 2008 ▶; Schmalz *et al.*, 1996 ▶; Ruther *et al.*, 1997 ▶; El-Kholi *et al.*, 2000 ▶). In a study by Schmalz *et al.* (1996 ▶), depression of the glass-transition temperature to as low as *T*_g_ ≃ 323 K was observed in samples of PMMA irradiated by X-ray synchrotron radiation (deep-etch regime) with a total exposure of ∼5.04 × 10^6^ Gy (converted from 6 kJ cm^−3^ in the energy density dose representation used in that work). In the standard chart of radiation tolerance of thermoplastic resins (Holmes-Siedle & Adams, 1994 ▶) this dose corresponds to the ‘destruction condition’. In another study direct melting of PMMA under soft X-ray synchrotron radiation was observed only after being exposed to 1.7 × 10^7^ Gy (El-Kholi *et al.*, 2000 ▶). Discrepancies in the damage threshold for PMMA may be related to the particular preparation of a sample (Zhang *et al.*, 1995 ▶), differences in molar masses and chemical bonding of polymer samples (Schmalz *et al.*, 1996 ▶), and different experimental conditions. It has been shown, for example, that cryo-cooling may prevent the mass loss of PMMA due to the diminishing mobility of reaction products under low-temperature conditions, although it does not influence damage related to photochemical reactions (*i.e.* bleaching and scissions; Beetz & Jacobsen, 2003 ▶; Coffey *et al.*, 2002 ▶). Calculation of the dose deposited per second in the PMMA sample using equation (12) for our experimental parameters gives a relatively high deposition rate of 1.02 × 10^2^ Gy s^−1^. For this rate, the ‘moderate–severe damage conditions’ of PMMA resist [∼8 × 10^4^ Gy, as tabulated by Holmes-Siedle & Adams (1994 ▶)] are attained after 8–12 min of exposure, when PMMA samples become noticeably deformed. However, this value is much less than, for example, the ‘melting transition dose’ observed by El-Kholi *et al.* (2000 ▶).

Such dramatic lowering of the ‘melting’ threshold dose in our experiments may be explained through an interplay of nanosize effects, *i.e.* lower stability due to the increased surface/volume ratio and cohesive interactions with support (Keddie *et al.*, 1994 ▶; Forrest & Dalnoki-Veress, 2001 ▶; Moller *et al.*, 1998 ▶; Rittigstein & Torkelson, 2006 ▶), and increased effective dose at the interface between PMMA samples and a Si_3_N_4_ support due to photoelectrons ejected from the relatively thicker support material, which causes additional damage in PMMA *via* the secondary electron cascades. This reasoning is based on a number of experimental and theoretical studies showing that there is an increased energy deposition region between a polymer resist and a support material with a higher absorption coefficient (Griffiths *et al.*, 2005 ▶; Pantenburg & Mohr, 1995 ▶; Schmidt *et al.*, 1996 ▶; Ting, 2003 ▶; Zumaque *et al.*, 1997 ▶). The calculated thickness of this interface layer in PMMA is around 1 µm (Ting, 2003 ▶) for a metalized support, which is much larger than the thickness of the PMMA sample in our experiments. Bulk Si_3_N_4_ has a higher X-ray absorption coefficient and a lower emissivity, ∊ ≃ 0.2 at ambient conditions compared with ∊ ≃ 0.92 in PMMA (Table 1 ▶). These properties may lead to a higher heat loading in nanopatterns of PMMA deposited on a silicon nitride membrane compared with stand-alone PMMA membrane samples.

### 6.2. Damage mechanisms in kapton–gold nanosample

It can be expected that for a collection of metallic nano­structures, such as gold nanospheres, the interface effects will be enhanced owing to their higher photoelectron yield (Schmidt-Ott *et al.*, 1980 ▶; Lewinski *et al.*, 2009 ▶), higher photoabsorption cross section and electron re-scattering from the surfaces of surrounding particles. Indeed, enhanced energy deposition properties of gold nanoparticles have been observed in studies on X-ray mediated damage in proteins (Brun, Duchambon *et al.*, 2009 ▶) and DNA (Carter *et al.*, 2007 ▶; Brun, Sanche *et al.*, 2009 ▶) in solution.

In our case, the confinement of gold nanoparticles in a narrow kapton gap could result in enhanced energy deposition owing to re-scattering of secondary electrons at the particle boundaries, which drives surface melting (Nanda *et al.*, 2007 ▶). A reduced thermal conductivity owing to porosity of the sample may lead to a higher rate of heat accumulation. Radiation heat transfer in a collection of gold nanoparticles dispersed in the gap between insulating kapton sheets was not included in our heat-transfer simulations. However, it was established that in such systems (‘nanoparticle beds’) the temperature growth rate owing to thermal radiation grows with porosity (Coquard & Baillis, 2005 ▶).

From the above discussion it is clear that the destruction of gold nanoparticles by synchrotron radiation is a complex multiscale process. To elucidate these mechanisms, a detailed investigation is needed which includes theoretical simulations, calorimetric control and spectroscopic measurements for monitoring the chemical and physical state of materials.

Modern models of phase transitions in irradiated materials utilize multiscale approaches, which combine Monte Carlo simulations of event cascades and molecular dynamics with a consideration of electronic excitation, electron–phonon and radiation transfer effects, equations of state, hydrodynamic simulations and thermodynamic analysis (Race *et al.*, 2010 ▶; Mao *et al.*, 2007 ▶; Duffy *et al.*, 2009 ▶; Bjorkas & Nordlund, 2009 ▶; Inogamov *et al.*, 2010 ▶; Francoeur *et al.*, 2008 ▶; Fu *et al.*, 2005 ▶; Phillips & Crozier, 2009 ▶; Lin *et al.*, 2008 ▶; Sanchez & Menguc, 2008 ▶). In the case of synchrotron-radiation-induced melting of materials, a combination of the two-temperature model (TTM; Anisimov & Luk’yanchuk, 2002 ▶) with Monte Carlo simulations of X-ray energy deposition represents one of the most promising approaches (Han *et al.*, 2002 ▶).

### 6.3. Role of heat sinks

Our numerical simulations of heat-transfer dynamics in composite nanosamples illustrate the importance of experimental conditions in CDI imaging at the nanoscale, *i.e.* the role of substrates, convection cooling and presence of heat sinks. A sample consisting of gold nanospheres dispersed between kapton sheets presents a good insulation material (*e.g.* composites from alternating thin layers of kapton and highly conducting metals, such as silver or gold, are used for thermal insulation of spacecrafts). In contrast, the previous studies of *in situ* growth of AlCu nanoparticles embedded in a single-crystal Al matrix (Zatsepin *et al.*, 2008 ▶) showed that the sample temperature jumped from 298 to 313 K within seconds of exposure to the X-ray beam. In that experiment the sample was an Al plate a few hundred micrometers thick, which was placed in a highly heat-conductive brass sample holder and the measurements were performed on samples already annealed at 493 K. The dose deposition rate was very high and reached the Henderson limit in approximately 5 s. However, the AlCu samples still showed structural integrity and produced stable diffraction patterns recorded during prolonged measurements (tens of hours). Heat losses through the brass sample holder may have reduced the radiation heat load in this experiment, thus supporting our argument that for metallic samples at relatively low X-ray energies the ‘classical’ heat-transfer mechanisms can be very significant to preserve the structural integrity of the specimens. It is also possible that annealing had increased the sample stability (Mei & Lu, 2007 ▶).

## 7. Progress in CDI using focusing optics

In CDI experiments the lowered thermodynamic stability of nanostructures can seriously limit their resolution owing to lower dose thresholds (Robinson, 2008 ▶; Marchesini *et al.*, 2003 ▶). However, a number of successful imaging experiments of gold nanosamples with a resolution of ∼50 nm have been reported recently (Marchesini *et al.*, 2003 ▶; Williams *et al.*, 2003 ▶, 2006 ▶; Pfeifer *et al.*, 2006 ▶). Unfortunately, some of the papers do not describe the beam intensity, temperature and ambient gas concentrations, as well as the sample damage after the experiments (Marchesini *et al.*, 2003 ▶; Williams *et al.*, 2003 ▶, 2006 ▶), which complicates the comparison of the damage thresholds.

On the other hand, the use of X-ray focusing optics to create high-energy density fluxes of coherent X-ray radiation has enabled a recent breakthrough in hard X-ray diffraction microscopy. The spatial resolution on a sub-10 nm spatial scale has been obtained in two (Schroer *et al.*, 2008 ▶; Takahashi *et al.*, 2009 ▶) and three dimensions (Takahashi *et al.*, 2010 ▶).

A successful high-resolution CDI imaging of a single 50 nm gold nanoparticle placed on a thin Si_3_N_4_ membrane was recently reported (Schroer *et al.*, 2008 ▶). The flux density on the sample exceeded 2.5 × 10^17^ photon s^−1^ cm^−2^ with a photon energy of 15.25 keV. The diffraction pattern was recorded in a series of ten 1 min exposures (total exposure 600 s). All measurements have been carried out in air at ambient temperatures. Judging by the diffraction patterns, at the above flux density the sample was stable, so the ten consecutive diffraction patterns and many others taken before were the same. However, after increasing the flux by an order of magnitude (pre-focusing), strong variations in the diffraction patterns were observed (C. G. Schroer, personal communication).

Similarly, in hard X-ray diffraction imaging experiments carried out at synchrotron beamline BL29XUL in SPring-8, the use of Kirkpatrick–Baez optics created a highly focused radiation flux, which allowed nearly diffraction limited imaging of a single silver 100 nm nanocube (Takahashi *et al.*, 2009 ▶) and a single 150 nm hollow Au/Ag nanobox (Takahashi *et al.*, 2010 ▶), which were placed on a thin, 100 nm-thick Si_3_N_4_ membrane support. These experiments were conducted in a vacuum chamber. The exposure times for small and high incident angles ranged from 100 to 800 s in Takahashi *et al.* (2009 ▶) and from 250 to 1650 s in Takahashi *et al.* (2010 ▶), respectively. The flux densities around the focal point were estimated to be ∼1.0 × 10^4^ photons nm^−2^ s^−1^ (Takahashi *et al.*, 2009 ▶) and 3.4 × 10^3^ photons nm^−2^ s^−1^ (Takahashi *et al.*, 2010 ▶).

To the best of our knowledge, a post-experimental examination of the samples in these experiments (Schroer *et al.*, 2008 ▶; Takahashi *et al.*, 2009 ▶, 2010 ▶) was not performed. Nevertheless, a comparative analysis of the results from these papers allowed us to draw some conclusions on the effects of the dose deposition rate, exposure and thermal insulation in our experiments.

Firstly, while in our experiments the dose deposition rate was lower, the resulting accumulated dose was higher owing to the continuous sample exposure. In the X-ray microscopy experiment (Schroer *et al.*, 2008 ▶) the dose was delivered by a series of short (1 min) exposures, which could have resulted in a better cooling of the sample between the exposures. Secondly, the heat-conduction properties of prismatic (Schroer *et al.*, 2008 ▶) or 100 nm-thick (Takahashi *et al.*, 2009 ▶, 2010 ▶) silicon nitride supports provided a much more efficient heat sink compared with the two relatively thick kapton sheets used in our experiments. Finally, since our sample had a high density of nanospheres, the photoelectron re-scattering between neighboring nanoparticles could also contribute to the damage. It has been noted that imaging of a single crystal may help to increase the dose threshold because photoelectrons are allowed to escape from the sample more easily (Cowan & Nave, 2008 ▶). It is estimated that in many materials of interest the mean path length of the secondary electrons generated by X-ray/XUV radiation can be as long as a few tens of nanometers (Ziaja *et al.*, 2006 ▶). This may reduce the radiation damage in single nanocrystals, since a significant portion of the energy could leave the crystal carried by the high-energy electrons escaping through the boundary of the crystal (Nave & Hill, 2005 ▶). However, in heterogeneous materials with different conductive and radiation-absorbing properties, the collision effects owing to the impacts of secondary electron cascades on the grain boundaries may lead to enhanced heat dissipation, atomic diffusion through the boundary and melting (Khorsand* et al.*, 2010 ▶).

## 8. Ultrashort pulse imaging using fourth-generation sources

The results of recent hard X-ray diffraction microscopy experiments suggest that the total number of photons, ∼1.5 × 10^11^, is required for a successful high-resolution structure reconstruction of a 100 nm nano-object. This is close to the peak photon flux per pulse of the Japanese XFEL facility (Takahashi *et al.*, 2009 ▶). Proposals for high-resolution structure determination schemes using XFEL are based upon the idea that it will be possible to collect enough information before disintegration of the samples. Simulations show that the onset of the structural damage, starting from the core-electron hole creation, followed by ionization-driven, plasma-like expansion and eventuating in the Coulomb explosion, becomes noticeable at 5−10 fs after the beginning of the exposure (Neutze *et al.*, 2000 ▶; Jurek *et al.*, 2004 ▶; Hau-Riege *et al.*, 2005 ▶). Owing to the extreme brightness of the highly coherent XFEL radiation, this would ideally happen after all the scattered radiation needed for successful reconstruction of the sample or its two-dimensional projection is collected at the detectors. Indeed, recent theoretical and experimental studies strongly support coherent diffraction imaging schemes utilizing ultra-intense, ultra-short-pulsed XFEL radiation to achieve a few-nm resolution of unique structures (Chapman *et al.*, 2006 ▶; Bogan *et al.*, 2008 ▶, 2010 ▶). For the regimes accessible by the X-ray free-electron laser in Hamburg (FLASH), it was shown that the onset of structural damage depends on both the radiation field intensity and wavelength, and the material properties of the sample and its size (Hau-Riege, London *et al.*, 2007 ▶, 2010 ▶). According to the experiments on multilayered optics (Hau-Riege, Chapman *et al.*, 2007 ▶), the nanostructures exposed to 25 fs pulses with flux values up to 3 × 10^14^ W cm^−2^ maintained their integrity and showed no structural changes over spatial scales exceeding 3 Å. Hydrodynamic modeling and experimental studies show that coating the imaged structures with a silicon sacrifice tamper layer can be effective in slowing down the sample’s expansion during repeated or prolonged pulse exposure, thus ‘making <1 nm resolution imaging feasible’ (Hau-Riege *et al.*, 2010 ▶).

After the Linear Coherent Light Source came into operation, a series of important ‘proof of principle’ experiments on imaging of biological objects (Seibert *et al.*, 2011 ▶; Chapman *et al.*, 2011 ▶) has been conducted at the AMO experimental station (Bozek, 2009 ▶). In one of these experiments, individual giant mimivirus particles (viral capsid size ≃ 0.45 µm) were injected into the pulse train characterized by 1.8 keV (6.9 Å) X-ray energy with the peak power density ∼6.5 × 10^15^ W cm^−2^ and pulse lengths estimated as ∼70 fs (full duration at half-maximum). The mimivirus particles underwent a hydrodynamic expansion on the picosecond timescale after exposure to the pulse, and were eventually evaporated; however, the recorded diffraction patterns were ‘exceptionally clean’ (Seibert *et al.*, 2011 ▶). This suggests that the structural changes at the attained spatial resolution scale of tens of nanometers were negligible during the 70 fs pulses. This experiment represents an important ‘stepping stone’ in developing the coherent diffraction imaging technique of the whole biological cells using fourth-generation sources. The resolution in these experiments may be greatly improved by using much higher intensity beams with a shorter wavelength (∼1.5 keV) and pulse lengths <5 fs combined with optimization of the detection and injection techniques (Seibert *et al.*, 2011 ▶). Averaging of multiple images of identical objects represents another possibility to improve the resolution.

In a second experiment, the method of ‘serial nanocrystallography’ for macromolecule structure determination was demonstrated while utilizing the same fluxes and energies (1.8 keV and 6.5 × 10^15^ W cm^−2^), with series of beams with 10, 70 and 200 fs pulse lengths (Chapman *et al.*, 2011 ▶). The diffraction peaks were collected from the injected fluid containing ∼1 mg ml^−1^ nanocrystals of Photosystem I with crystal sizes ranging from 200 nm to 2 µm and combined into a final set of three-dimensional structure factors. The stream was intercepted by 70 fs pulses with a 30 Hz pulse rate, which produced final structures with a resolution of 8.5 Å. It was found that the properties of the integrated Bragg intensities generated by 10 and 70 fs pulses were similar, so there were no signatures of radiation damage for the given resolution. However, the longer pulses (200 fs) caused radiation damage which resulted in poor resolution beyond 25 Å. It is anticipated that at 1.5 Å wavelength shorter pulses with higher repetition rates will allow for a more efficient data collection. In combination with novel phasing and indexing algorithms this will provide a basis for a near-atomic resolution imaging (Chapman & Nugent, 2010 ▶). The serial nanocrystallography utilizing symmetry-adapted indexing and averaging of the multiple diffraction patterns of the hydrated membrane protein nanocrystals collected ‘on the fly’ will enable studies of such miniscule amounts of material without the need for cryocooling, which are not possible to study by means of conventional crystallography (Hunter *et al.*, 2011 ▶; Caleman *et al.*, 2011 ▶; Saldin *et al.*, 2011 ▶; Kirian *et al.*, 2010 ▶, 2011 ▶).

However, imaging of a single macromolecule with atomic resolution might require even shorter pulses and a few-fold increase in power. For very intense fields, the problem of electronic density damage may impose a sub-femtosecond limit on the pulse length. In view of the latest experimental results (Young* et al.*, 2010 ▶; Berrah* et al.*, 2010 ▶), the conventional rates of quantum dynamical processes such as ionization and Auger processes may need to be reassessed for the high-intensity X-ray fields. XFEL fields give rise to novel electron transition resonances within individual atoms, such as the creation of ‘transparent’ hollow atoms which show increased stability against Auger deterioration (Son *et al.*, 2011 ▶). Simulations show that the ultra-intense fields may stimulate a nonlinear spatial transport of electronic charge in individual molecules *via* coherent tunneling between nuclear centres (Ponomarenko, 2011 ▶), plasma effects (Saalmann, 2010 ▶) and a combination of these effects producing charge-density solitons in a gaseous sample (Fratalocchi & Ruocco, 2011 ▶).

## 9. Conclusions

While the spatial resolution in a diffraction-imaging experiment is inversely proportional to the radiation energy, the linear absorption coefficient away from the absorption edge decreases approximately as the inverse-square of the photon energy. Therefore, an increase in the radiation energy would result in both higher resolution and lower heat load. However, the diffraction contrast is inversely proportional to the real part of the sample’s refractive index so that it decreases as approximately the inverse-square of the energy increase. While low-resolution (50–100 nm) imaging of thin organic structures is possible (Chapman *et al.*, 2006 ▶), a high-resolution (<1–5 nm) imaging of ultra-thin (<50 nm) nanostructures requires extreme care (Nikulin *et al.*, 2008 ▶). Our results demonstrate the high sensitivity of material structure nanosamples to particular conditions during the synchrotron coherent diffraction imaging experiments.

There are several ways to optimize the CDI experimental conditions utilizing synchrotron radiation. First it might be possible to decrease the dose rates by lowering both the beam intensity and the photon energy without losing resolution (Marchesini *et al.*, 2003 ▶). Second, enhanced convection or cryocooling of the sample (*e.g.* cryoloops) seems to decrease the damaging effects so that serious considerations should be given to providing an adequate heat exhaust from the samples that undergo X-ray microscopy or diffractive imaging. Third, the choice of support seems to be very important. While the silicon nitride membranes are frequently used in imaging of nanosamples, other X-ray-transparent, highly heat-conducting, low-*Z* materials such as Be should also be tested. Furthermore, splitting the exposure time into a series of short pulses may also be applied to decrease the heat-loading effects. This issue is even more important in the case of CDI with a focused beam (Quiney *et al.*, 2006 ▶).

Some new algorithms have also been proposed to circumvent the damage problem in the XFEL structure determination experiments. These include:

(i) The symmetry-adapted indexing and averaging of the multiple diffraction patterns of the nanocrystals collected ‘on the fly’ combined with Bayesian refinement (Fung *et al.*, 2009 ▶), and

(ii) The algorithm based on the use of the maximally complete information on the quantum dynamics of the electronic shell configurations interacting with the probe field to reconstruct positions of nuclear centers in a single macromolecule (Quiney & Nugent, 2011 ▶).

In contrast to XFEL imaging experiments, the regimes of photon energy deposition in third-generation synchrotron sources are rather different. Even in the hard X-ray diffraction microscopy experiments with focusing mirrors, the total number of photons required for a high-resolution nano­structure reconstruction is collected over many minutes of exposure. The processes leading to the conversion of absorbed photon energy into the thermal energy of vibrating atomic lattice happen on the picosecond scale. In optimized coherent-diffraction imaging experiments using synchrotron sources the effects of heat loading owing to absorbed radiation cannot be ignored. This necessitates a detailed analysis of exposure regimes, spatial scales of nanostructures and their features, ambient gas conditions and properties of the support material as factors affecting the damage thresholds levels. We believe that the benefits of such investigations would expand well beyond the coherent-diffraction imaging methods to a much broader field that covers the application of intense synchrotron radiation for material processing.

## Figures and Tables

**Table 1:** Material-dependent parameters used in numerical simulations of heat transfer

Material	Density ρ (kg m^−3^)	Specific heat *c*_p_ (J kg^−1^ K^−1^)	Thermal conductivity κ (W m^−1^ K ^−1^)	Attenuation (λ = 0.1 nm) μ (m^−1^)	Emissivity ∊
Gold (bulk)	19290^*a*^	129^*b*^	310^*b*^	317500.60^*a*^	0.0001–0.3†
Kapton	1430^*a*^	1090^*c*^	0.385^*c*^	222.75^*a*^	0.0001–0.95†
Si_3_N_4_	3440^*a*^	700^*d*^	25^*d*^	3772.67^*a*^	0.2^*e*^
Si	2329^*a*^	700^*f*^	148^*f*^	3968.30^*a*^	0.7^*g*^
PMMA	1190^*a*^	1220–2170^*h*^	0.2^*i*^–0.3^*j*^	197.44^*a*^	0.92^*k*^
Air (ambient)	1.177^*l*^	1.006^*l*^	0.026^*l*^	–	–

The simulation parameters are taken from (*a*) CXRO (2005 ▶) and Henke *et al.* (1993 ▶); (*b*) Weast (1974 ▶); (*c*) McAlees (2002 ▶); (*d*) ATC (2010 ▶); (*e*) Völklein (1990 ▶); (*f*) Sikora (2010 ▶); (*g*) Ravindra *et al.* (2003 ▶); (*h*) Soldera *et al.* (2010 ▶) and Hempel *et al.* (1996 ▶); (*i*) Assael *et al.* (2008 ▶) and Rudtsch & Hammerschmidt (2004 ▶); (*j*) Wen *et al.* (1993 ▶); (*k*) Baek *et al.* (1997 ▶); (*l*) Holman (2002 ▶).

† In the simulations a wide range of emissivity values for gold and kapton outer layers were tested to account for the different heat radiation regimes. The values of kapton emissivity depend on the state of its outer surface (rough or smooth), thickness and backing material, so a few micrometers thick metalized films have very small emissivity (∼0.03; SNAP, 2003 ▶). The standard values for thicker kapton varieties are 0.54 (SNAP, 2003 ▶), 0.78–0.84 (McAlees, 2002 ▶). The measured emissivity of thin gold films, ∊ = 0.3 (for 1.0 µm thickness), 0.01–0.1 (for 1.6 µm thickness; Raytek, 2000 ▶).
